# Reestablishment of perfusion in critical limb ischemia model with pulsed focused ultrasound (pFUS) and mesenchymal stem cells in aged mice

**DOI:** 10.1186/2050-5736-3-S1-P105

**Published:** 2015-06-30

**Authors:** Pamela Tebebi, Victor Frenkel, Rashida Williams, Scott Burks, Joseph Frank, Ben Nguyen, Saejeong Kim

**Affiliations:** 1 National Institutes of Health, Bethesda, Maryland, United States; 2 University of Maryland School of Medicine, Baltimore, Maryland, United States

## Background/introduction

Severe chronic peripheral arterial disease (PAD) manifests as critical limb ischemia (CLI) with a 5-year mortality rate >70%. Mesenchymal stem cells (MSC) show promise to minimize CLI progression and restore perfusion in experimental models, but approaches suffer from minimal homing to ischemic tissue and require clinically impractical direct injections of cells. We show that pulsed focused ultrasound (pFUS) noninvasively establishes a “molecular zip-code” of locally upregulated chemoattractants (i.e. cytokines, chemokines, cell adhesion molecules) that lead to enhanced homing permeability and retention of IV-infused MSC to pFUS-treated muscle. This study investigated if pFUS could enhance MSC homing in a CLI model in aged mice and whether they could ultimately improve limb perfusion.

## Methods

CLI was induced by ligating the external iliac artery in C3H mice (9-12 months old). At 14 days post-CLI, mice were grouped as followed: saline controls (n=8); pFUS alone (n=7), MSC alone (n=8), or MSC+pFUS (n=17). Mice were treated, according to their group, daily for 3 days (i.e, 3×pFUS, 3×MSC, 3×MSC+pFUS). pFUS was applied to ischemic hamstrings at 40W (5% duty cycle, 5Hz PRF, 100 pulses). For mice receiving MSC, 106 MSC were IV injected via the lateral tail vein. Mice receiving pFUS+MSC had MSC injected ~1hr pre-pFUS. To assess MSC homing, mice were euthanized at 15 days post-CLI (24 hr after last treatment). Fe-labeled MSC were counted by microscopy after Prussian Blue staining. Laser Doppler perfusion imaging (LDPI) was performed weekly for 7-8 weeks post-treatment.

## Results and conclusions

**Figure 1:**
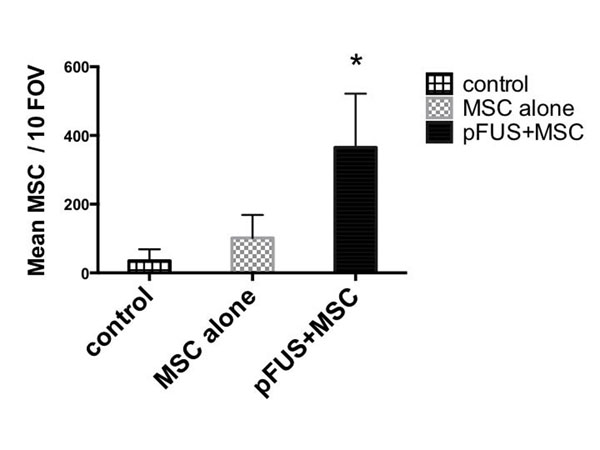
14 days post-CLI, mice were given either MSC alone or pFUS+MSC and hamstrings were harvested 24 hrs. MSC failed to home to ischemic limbs without pFUS, but significantly more (p<0.05) MSC homed to ischemic limbs when they were treated with pFUS.

**Figure 2:**
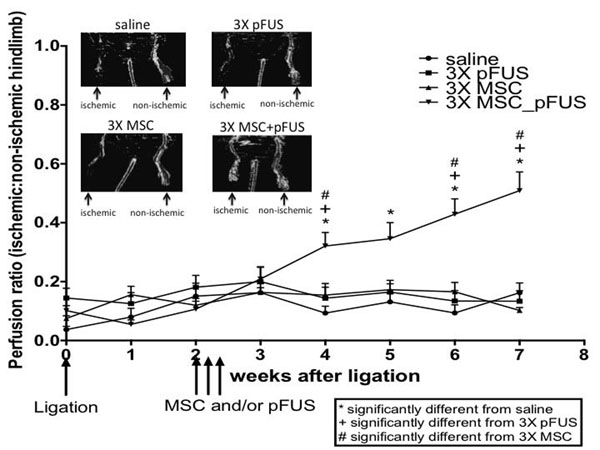
14 days after CLI induction mice received 3 consecutive days of pFUS, MSC, or MSC+pFUS. At 2 weeks post treatment significant increase (p<0.05) in laser Doppler blood flow compared to 3XpFUS, 3XMSC, and saline group.

Results: Significantly more MSC homed to pFUS-treated ischemic hamstrings than ischemic hamstrings without pFUS (p<0.01) or normal hamstrings (Fig 1). LDPI revealed that restoration of limb perfusion was significantly greater in the pFUS+MSC mice beginning 2 weeks post-treatment (4 weeks post-ischemia) (Fig 2). Limb perfusion in the pFUS+MSC group continued to improve for the remainder of the study (7 weeks post-ischemia), while groups treated with MSC alone, pFUS alone, or saline remained near baseline perfusions levels. Conclusion: pFUS-enhanced MSC homing is a clinically relevant modality with potential to treat PAD. Previous experimental CLI studies have shown promise, but they inadequately model clinical PAD. They utilize direct cell injections into ischemic muscle, administer cells immediately after injury, and use young animals with robust healing capabilities. We show that when treatment is delayed (14 days post-CLI, during sub-acute inflammation) in aged mice, iv-injected MSC largely fail to home to ischemic muscle and have limited therapeutic potential. However, pFUS can noninvasively establish a molecular zip-code of upregulated chemoattractants to enhance MSC homing where therapeutic MSC can help restore perfusion to ischemic limbs. As direct injections are impractical clinically, systemic MSC delivery is necessary and pFUS guidance of MSC may be critical to develop effective cell therapies in PAD.

